# Glanders Diagnosis in an Asymptomatic Mare from Brazil: Insights from Serology, Microbiological Culture, Mass Spectrometry, and Genome Sequencing

**DOI:** 10.3390/pathogens12101250

**Published:** 2023-10-17

**Authors:** Paula Adas Pereira Suniga, Cynthia Mantovani, Maria Goretti dos Santos, Andréa Alves do Egito, Newton Valério Verbisck, Lenita Ramires dos Santos, Alberto Martín Rivera Dávila, Cristina Kraemer Zimpel, Maria Carolina Sisco Zerpa, Daniela Pontes Chiebao, Ana Márcia de Sá Guimarães, Alessandra Figueiredo de Castro Nassar, Flábio Ribeiro de Araújo

**Affiliations:** 1 Postgraduate Program in Animal Science, Faculty of Veterinary Medicine and Animal Science-FAMEZ/UFMS, Federal University of Mato Grosso Do Sul, Av. Senador Filinto Muller, 2443, Campo Grande 79074-460, MS, Brazil; paula_adas@hotmail.com (P.A.P.S.); andrea.egito@embrapa.br (A.A.d.E.); 2 MAI/DAI Scholarship, Federal University of Mato Grosso Do Sul, Cidade Universitária, Av. Costa E Silva, Campo Grande 79070-900, MS, Brazil; 3 Embrapa Beef Cattle/Ministry of Agriculture and Livestock Scholarship, Embrapa Beef Cattle, Av. Rádio Maia, 830, Campo Grande 79106-550, MS, Brazil; cymant@hotmail.com; 4 Embrapa Beef Cattle, Campo Grande 79106-550, MS, Brazil; goretti.santos@embrapa.br (M.G.d.S.); newton.verbisck@embrapa.br (N.V.V.); flabio.araujo@embrapa.br (F.R.d.A.); 5 Computational and Systems Biology Laboratory, Graduate Program in Biodiversity and Health, Oswaldo Cruz Institute, Fiocruz, Rio de Janeiro 21040-900, RJ, Brazil; amrdavila@gmail.com; 6 Department of Microbiology and Molecular Genetics, Michigan State University, East Lansing, MI 48824, USA; kraeme19@msu.edu; 7 Laboratory of Applied Research in Mycobacteria, Department of Microbiology, Institute of Biomedical Sciences, University of São Paulo, São Paulo 05508-000, SP, Brazil; carolsisco@gmail.com (M.C.S.Z.); anamarcia@usp.br (A.M.d.S.G.); 8 Animal Health Research Center, Biological Institute, Av. Conselheiro Rodrigues Alves, 1252, São Paulo 04014-002, SP, Brazil; daniela.chiebao@sp.gov.br (D.P.C.); afcnassar@sp.gov.br (A.F.d.C.N.)

**Keywords:** *Burkholderia mallei*, genome, glanders

## Abstract

This manuscript elucidates the occurrence of glanders in an asymptomatic mare from Brazil presenting positive *Burkholderia mallei* antibody titers. The diagnosis was established through a multi-pronged approach encompassing microbiological culture, mass spectrometry, and genome sequencing. The outbreak occurred in 2019 in Tatuí, São Paulo, Brazil, and the infected mare, despite displaying no clinical symptoms, had multiple miliary lesions in the liver, as well as intense catarrhal discharge in the trachea. Samples were collected from various organs and subjected to bacterial isolation, molecular detection, and identification. The strain was identified as *B. mallei* using PCR and confirmed by MALDI-TOF mass spectrometry. Whole-genome sequencing revealed a genome size of 5.51 Mb with a GC content of 65.8%, 5871 genes (including 4 rRNA and 53 tRNA genes), and 5583 coding DNA sequences (CDSs). Additionally, 227 predicted pseudogenes were detected. In silico analysis of different genomic loci that allow for differentiation with *Burkholderia pseudomallei* confirmed the identity of the isolate as *B. mallei*, in addition to the characteristic genome size. The BAC 86/19 strain was identified as lineage 3, sublineage 2, which includes other strains from Brazil, India, and Iran. The genome sequencing of this strain provides valuable information that can be used to better understand the pathogen and its epidemiology, as well as to develop diagnostic tools for glanders.

## 1. Introduction

*Burkholderia mallei* is a Gram-negative bacterium that causes glanders, a contagious and potentially fatal disease in equids such as horses, mules, and donkeys [1]. It can also infect other animal species, including humans [2], particularly those in close contact with infected equids. Apart from its pathogenicity in animals, *B. mallei* is also a significant concern for public health due to its potential as a bioterrorism agent [3]. The CDC has listed the bacterium as a Category B bioterrorism agent, indicating its potential for significant public health impact [4].

The World Organization for Animal Health (WOAH) recognizes four clinical presentations of glanders, including nasal, pulmonary, cutaneous, and asymptomatic carrier forms. The nasal form is characterized by the development of inflammatory nodules and ulcers in the nasal passages, leading to a sticky yellow discharge and stellate scarring during the healing process. The pulmonary form results in nodular abscesses in the lungs, progressive weakness, coughing, and diarrhea, while the cutaneous form (also known as “farcy”) causes the enlargement of lymph vessels and nodular abscesses along their course, which can ulcerate and produce yellow pus. Nodules in the liver and spleen can lead to wasting and eventual death [5].

In Brazil, from 1999 to 2022, 3385 cases of glanders were reported. Of these, 715 cases occurred in the last 3 years, according to reports from the Brazilian Ministry of Agriculture and Livestock [https://indicadores.agricultura.gov.br/saudeanimal (accessed on 5 October 2023)]. This may be partly attributed to the use of more sensitive and specific diagnostic methods for glanders diagnosis compared to the complement fixation test [6].

In Brazil, equids that work in sugar cane fields in the forest zone of the Northeast are more frequently reported to exhibit symptomatic cases of glanders [7,8,9]. Conversely, reports on glanders in other regions generally describe asymptomatic animals or those with mild clinical changes [10,11]. However, there has been no systematic study to evaluate the comprehensiveness of the clinical and physical examinations that have led to the determination of asymptomatic status in these animals.

Diagnostic tests for glanders worldwide heavily rely on serology. In Brazil, the previous law (Normative Instructions nr. 6, 2018) mandated euthanasia for seropositive equids. However, relying solely on serology as the diagnostic criterion, especially in asymptomatic horses, has caused distrust within the equine sector. Consequently, legal disputes arose when horse owners opposed mandatory euthanasia.

Recently, the legislation in Brazil has changed (Portaria MAPA nr. 593, 2023) and suspected cases of glanders are identified as equine that are susceptible and show clinical or pathological signs compatible with glanders or when there is an epidemiological link to a confirmed outbreak or case. Confirmation of cases involves isolating and identifying *B. mallei* in a sample from an equine or detecting specific antigens, genetic material, or antibodies related to *B. mallei* in a sample from an equine displaying clinical or pathological signs compatible with glanders.

There is an increasing need to isolate and characterize Brazilian strains of *B. mallei* to better comprehend its transmission and virulence aspects [10,11,12]. This paper aims to illustrate the detection of *B. mallei* in a seropositive mare that displayed no clinical symptoms, employing a comprehensive approach that integrates microbiological culture, mass spectrometry, and genome sequencing.

## 2. Materials and Methods

### 2.1. Clinical Case

**Figure 1:**
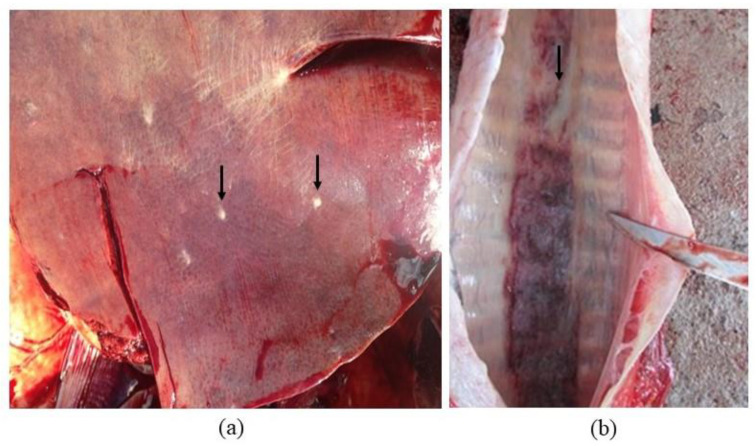
Necropsy findings (arrows) from a mare without clinical signs. (**a**) Multiple miliary lesions in the liver. (**b**) Intense catarrhal discharge in the trachea.

In 2019, an outbreak of glanders in horses was identified in the city of Tatuí, São Paulo, Brazil, by state agricultural defense services. During the outbreak, one young mare was found to have a positive result in both the complement fixation (CF) screening test (cold procedure) and Western blot (WB), which were performed in an official laboratory according to the Brazilian Equine Health Program. In Brazil, the CF test employs antigens from the USDA, USA, but undergoes an in-house standardization process, resulting in variable test accuracy. Conversely, the WB test (Biovetech, Recife, Brazil) boasts a diagnostic sensitivity and specificity of 100%. Despite having a healthy body condition score and displaying no clinical symptoms, the mare was found to have multiple miliary lesions in the liver and intense catarrhal discharge in the trachea (see Figure 1) during necropsy. Additionally, the mediastinal lymph nodes were enlarged, and the spleen was hypoplastic. Secretion and/or tissue samples were collected from various organs, including the lungs, trachea, lymph nodes, heart, spleen, kidney, and liver.

### 2.2. Bacterial Isolation and Molecular Detection

All tissue samples were suspended in 0.85% sterile saline at a concentration of 1:5 *w*/*v*, and 10 µL of the suspension was plated on 5% sheep blood agar supplemented with 1% glycerin and 2500 IU of potassium benzylpenicillin. The plates were then incubated at 37 °C for 48–72 h [10,13,14]. The resulting bacterial colonies were examined for their morphological characteristics, including size, shape, color, and the presence and type of hemolysis. After Gram staining, the colonies were further examined for their cellular distribution and staining characteristics. Biochemical tests, including catalase, oxidase, indole, nitrate reduction, Voges-Proskauer test, motility, and fermentation of sugars, were also performed as described by Quinn et al. (2011) [13] and Winn et al. (2008) [14]. To confirm the presence of *B. mallei*, colonies were resuspended in 0.85% saline, inactivated at 100 °C for 10 min, and stored at −20 °C for molecular analysis. PCR was performed, targeting *fliP*-IS407 using primers described by Abreu et al. (2020) [10], which produced a 528 bp fragment (*fliP*-IS407 F: 5′ TCAGGTTTGTATGTCGCTCGG 3′ and *fliP*-IS407 R: 5′ GCCCGACGAGCACCTGATT 3′. A reference strain of *B. mallei* INCQS 00,115 (ATCC 15310) from the Collection of Reference Microorganisms in Sanitary Surveillance, FIOCRUZ-INCQS, Rio de Janeiro, Brazil, was used as a positive control, and sterile deionized water was used as a negative control.

### 2.3. MALDI-TOF Mass Spectrometry

Bacterial samples were inactivated with ethanol, and protein profiles were acquired and analyzed by MALDI Biotyper™ (Bruker Daltonics, Billerica, MA, USA), as described [15,16]. Briefly, isolated colonies were picked and washed twice with sterile water centrifuged for 1 min at 10,000× *g* at room temperature. The cell pellet was suspended in 300 µL of sterile water and inactivated by the addition of 900 µL absolute ethanol. After centrifugation at 10,000× *g* at room temperature for 2 min, bacterial proteins were extracted with 70% formic acid and pure acetonitrile. The supernatant containing proteins was collected after centrifugation at 10,000× *g* at room temperature for 2 min, and 1 µL was spotted in a clean stainless steel MALDI target (Bruker Daltonics, Billerica, MA, USA) in triplicate. Upon air drying, bacterial proteins were overlaid with 1 µL of a-cyano-4-hydroxy-cinnamic acid (5 mg/mL) matrix solution and allowed to dry in the air. Mass spectra were acquired with an Autoflex III Smartbeam (Bruker Daltonics, Billerica, MA, USA) in a mass to charge (*m*/*z*) range of 2000–20,000 Daltons after calibration with an *E. coli* standard (IVD BTS, Bruker Daltonics, Billerica, MA, USA), as described [17]. For bacterial identification, mass spectra were processed with MALDI Biotyper™ v.3.1 (Bruker Daltonics, Billerica, MA, USA) using the standard method and identification criteria with MBT Compass Library DB-7311 v.7.0.0.0 (Bruker Daltonics, Billerica, MA, USA), containing 7311 mass spectra profiles (MSPs) for 434 genera and 2509 species of microorganisms. Since Bruker’s library DB-7311 lacks *B. mallei* and *B. pseudomallei* MSPs, we have included reference mass spectra for those species using *B. mallei* ATCC 23344 and *B. pseudomallei* mass spectra from Robert Koch Institut (RKI), Berlin, Germany, available at Zenodo [18], and MSPs for *B. mallei* ATCC 15310 and seven other *B. mallei* and *B. pseudomallei* clinical isolates previously characterized by our group [19]. The *B. mallei* MSP for the BAC 86/19 clinical isolate described in this work was generated with the MALDI Biotyper™ standard method after processing 30 mass spectra using 70 *m*/*z* signals with a frequency higher than 50%.

### 2.4. Whole-Genome Sequencing

The Whole Genome Sequence (WGS) analysis for BAC 86/19 was conducted at the NGS multiuser platform of Oswaldo Cruz Foundation (FIOCRUZ), located in Rio de Janeiro, Brazil. First, DNA quantification was performed using the Qubit™ dsDNA HS Assay Kit (Thermo Fisher Scientific, Waltham, MA, USA) and the Agilent High Sensitivity DNA Kit (Agilent, Santa Clara, CA, USA). The WGS procedure was carried out on a HiSeq instrument (Illumina, San Diego, CA, USA) using HiSeq Rapid SBS Kit v2 (200 cycles) chemistry and the Nextera DNA Flex Library preparation kit (Illumina, San Diego, CA, USA) following the manufacturer’s guidelines.

The resulting reads were deposited in SRA (Sequence Read Archive), NCBI (National Center for Biotechnology Information) under accession number SRR19621772.

### 2.5. Quality Assessment, Assembly, and Annotation

Reads were evaluated using FastQC [20] before and after trimming. Quality trimming was performed using Trimmomatic v. 0.39 (sliding window: 5:20) [21]. Quality-trimmed reads were assembled using SPAdes genome assembler v3.15.4 with default parameters [22]. The resulting contigs were organized into scaffolds using RagTag RagTag [23] using the reference genome of *B. mallei* ATCC 23344 (Accession numbers: CP000010.1 and CP000011.2). The assembled genome was deposited in NCBI and annotated with PGAP (Prokaryotic Genome Annotation Pipeline) (Accession number: JANCTE01).

### 2.6. Genetic Markers for Species Confirmation

Primers and probes used in real-time PCR assays to differentiate between *B. mallei* and *B. pseudomallei* were used to confirm the bacterial species of the isolate. Briefly, primer and probe sequences used to detect *orf11* and *orf13* genes (primers: PM122, orf11R, orf13f, orf13r; probes: orf11pro, orf13pro) [24], as well as the BMA10229_0375 [25] and *mprA*-associated regions (primers: 14F5, 14R5) [26], were searched against the sequencing reads of the isolate using the “filter” tool of the “Reads” tab of the SRA’s Run Browser of the deposited isolate’s reads. Only exact matches (i.e., 100% identity) can be identified using this tool, and when detected, the minimum number of reads observed with identical matches to the queried primers and probes was 48. In addition, the 23S rRNA and *serB* genes of the isolate sequenced herein were retrieved and subjected to a global alignment against the 23S rRNA and *serB* genes of the reference genome *B. pseudomallei* Mahidol-1106a (ASM75612v1; Accession numbers: NZ_CP008781.1 and NZ_CP008782.1).

### 2.7. Lineage Identification

The lineage of *B. mallei* BAC 86/19 was identified following a scheme previously reported by Girault et al. (2018) [27]. The scheme uses 15 phylogenetically informative SNPs to define 3 lineages and 12 sublineages. For this, quality trimmed reads of *B. mallei* BAC 86/19 were mapped to the reference genome *B. mallei* ATCC 23344 using BWA-MEM [28] with default parameters. Variant calling was performed using GATK version 4.1.9.0 [29,30], selecting a quality depth of 7, Fisher strand value of 60, mapping quality of 40, and strand odds ratio of 3. BaseCalibrator and ApplyBQSR tools were applied before the final variant call and filtration, and alleles were annotated using SnpEff version 5 [31]. All 15 genomic positions [27] were investigated in the vcf file to determine the base called and used to determine the lineage and sublineage.

## 3. Results

### 3.1. Bacterial Isolation and Molecular Detection

During necropsy, a total of eleven samples were collected. Among these, one sample from the tracheal secretion showed suggestive colonies of *B. mallei* growth on a selective blood-enriched medium, as previously described by Abreu et al. (2020) [10]. The colonies exhibited small, gray, shiny, and nonhemolytic characteristics. After conducting phenotypic identification, the isolate was further confirmed through fliP-IS407 PCR.

### 3.2. MALDI-TOF Mass Spectrometry

Strain BAC 86/19 was analyzed by MALDI-TOF MS, and identification at the species level was achieved after inclusion of its own MSP to the Biotyper™ library available locally (Supplementary Table S1). As a validation of our analysis, we used *B. mallei* and *B. pseudomallei* MSPs of reference from Zenodo.org [18] and from clinical strains isolated and provided by the Federal Agricultural Defense Laboratory of Minas Gerais, Brazil, previously characterized by our group [19]. After inclusion of the *B. mallei* BAC 86/19 MSP, Biotyper™ score values were above 2.3, consistent with reliable identification at the species level, which was observed in all 86/19 strain samples tested (Supplementary Table S1). It is noteworthy that *B. pseudomallei*, which is closely related to *B. mallei,* was frequently observed as the second-best match, with relatively lower scores.

### 3.3. The Draft Genome of B. mallei BAC 86/19

The sequencing of the bacterial isolate generated a total of 1,368,490 reads, which amounted to 260.6 Mbp with a coverage of approximately 47x. Genome assembly resulted in 2067 contigs, which were subsequently assembled into 48 scaffolds. The N50 of scaffolds was found to be 3,324,748 bp, with 2019 spanned gaps and a total ungapped length of 5,305,955 bp. The presence of repetitive sequences in the *B. mallei* genome has been known to result in a substantial number of contigs during genome assembly using short reads [32]. The estimated genome size was 5,507,851 bp. PGAP genome annotation revealed a GC content of 65.8%, 5871 genes (including 4 rRNA and 53 tRNA genes), and 5583 coding DNA sequences (CDSs). Additionally, PGAP predicted 227 pseudogenes.

### 3.4. Species Confirmation Using Genetic Markers

The genome was initially analyzed using Kaiju software v.1.9.0 [33], showing, showing that >97% of the reads were *Burkholderia* spp., with 12% of the reads specifically assigned to *B. mallei*. Krona [34] analyses also assigned > 99% of the reads to *Burkholderiaceae*. Therefore, the isolate was free of major bacterial contaminants (i.e., non-*Burkholderia* species) and an estimated genome size of 5.51 Mb was formed, a size consistent with expectations.

### 3.5. Lineage Identification

According to a scheme proposed previously [27], *B. mallei* BAC 86/19 was identified as lineage 3, sublineage 2 (Table 1).

## 4. Discussion

Glanders is a zoonosis that causes significant economic losses, especially when endemic. The characterization of the strains circulating in the countries is an important way to contribute to the development of strategies for sanitation and eradication of the pathogen. Bacterial isolation is a challenging factor reported in the literature, especially for chronic animals with lower pathogen loads in the tissues and higher microbial diversity [10,11].

In this study, an asymptomatic mare was diagnosed with glanders, initially relying on serology using CF as a screening test and WB as a confirmatory test. The World Organization for Animal Health (OIE) guidelines recommend that a diagnosis of glanders should be supported by positive serological test results. This typically involves an initial screening with CF, followed by confirmation through a second test with equal or higher sensitivity and higher specificity, such as the *B. mallei*-specific lipopolysaccharide (LPS) WB, among other recommended methods [5].

Recently, Brazilian legislation has introduced additional criteria for confirming glanders in seropositive animals. These criteria include the presence of clinical or pathological signs consistent with glanders, direct detection of *B. mallei* in microbiological cultures, or the identification of specific antigens or genetic material in biological samples. However, there is accumulating evidence supporting the high specificity of the serological tests currently utilized in Brazil, such as ELISA and WB, as documented by [11].

Bacterial colonies were chosen with characteristic morphology: round, grayish, non-hemolytic, and isolated. In addition, to characterize the bacteria of interest, biochemical tests were used. Previous studies in Brazil with a strain from the Brazilian Northeast reported small differences in the fermentation of some carbohydrates, but without compromising bacterial identification [35].

The differentiation of *B. mallei* from *B. pseudomallei* has been historically difficult due to their high genomic similarity [32]. A major phenotypic difference between the two species is motility. Due to the insertion of an *IS407A* element in the *fliP* gene, *B. mallei* lost its motility capacity. Accordingly, the *fliP* gene of the strain sequenced herein has been predicted by PGAP as a pseudogene (locus_tag = “LV178_14020”). Genetic events leading to pseudogenization are classified by PGAP as frameshift, internal stop codon or incomplete. The *fliP* pseudogene is described as incomplete, which agrees with a potential disruption caused by an insertion sequence. The genome size can also be used to distinguish *B. mallei* from *B. pseudomallei*; *B. mallei* has a smaller genome size than *B. pseudomallei* (~7.2 Mb) [32]. Accordingly, *B. mallei* BAC 86/19 showed an estimated genome size of 5.51 Mb, which is in accordance with other *B. mallei* strains and smaller than that of *B. pseudomallei* [32].

Many PCR assays have been developed to differentiate between *B. mallei* and *B. pseudomallei* [32]. Exact matches (100% sequence identity) to primer and probe sequences of the orf13 and BMA10229_0375 regions were identified among the sequenced reads, while exact matches to the primer and probe sequences of the orf11 and *mprA*-associated regions were not detected, which also confirms the isolated species as *B. mallei*. Finally, the global sequence alignment between the 23S rRNA and the *serB* genes of the sequenced strain with that of the reference genome of *B. pseudomallei* Mahidol-1106a also showed the presence of SNPs previously identified as specific to *B. mallei* [36,37].

According to Falcão et al. (2022) [38], the L3B2 lineage in Brazil consists of several isolates of *B. mallei*, including 9902 RSC, BM_campo 1, BM_campo 3, and UFAL2, which were obtained from the Northeast region of Brazil. Laroucau et al. (2018) also confirmed that the 16-2438_BM#8 strain, collected from the same region, is part of this lineage. Additionally, this lineage encompasses isolates from India (NCTC3708, NCTC3709) and Iran (A200) [27].

The L3B2 lineage in Brazil includes isolates that have caused either clinical infections (such as UFAL 2 and 16-2438_BM#8) or asymptomatic infections (such as BAC 86/19, 9902 RSC, BM_campo 1, and BM_campo 3) [38,39].

Due to the challenges involved in culturing *B. mallei*, such as the need for tissue collection and preservation, slow growth on media without glycerol supplementation, and the requirement of a biosafety laboratory, bacteriological analyses are not always feasible or successful [39]. To reduce the risks and difficulties of dealing with high-risk pathogens such as *B. mallei*, MALDI-TOF is a safe and fast alternative for accurate identification, due to its inactivation with ethanol, to extract proteins at a biosafety level [40].

The analysis of the 86/19 strain using MALDI-TOF involved its characterization across various culture media, resulting in species-level identification. This step effectively mitigated a previously acknowledged bias [41]. The process of sample preparation played a pivotal role in refining the spectral outcomes. Notably, the methodology yielding the most superior spectra quality involved the utilization of ethanol and formic acid. This choice stemmed from its enhanced protein extraction efficiency and its non-deleterious impact on storage conditions. Moreover, the incorporation of a specialized reference set, coupled with the acquisition of high-caliber spectra, enabled a clear-cut differentiation between the two distinct species [40].

Expanding the isolation of *B. mallei* in different regions of Brazil [11] will enable a comprehensive genomic characterization, generating knowledge about genetic diversity, transmission events, and genetic markers for different strains.

## Figures and Tables

**Table 1:** Lineage identification of *Burkholderia mallei* BAC 86/19 based on a scheme previously described (Girault et al., 2018 [27]).

Lineage	Genomic Position *	Reference Base to Be Called a Lineage **	Base in *B. mallei* BAC 86/19
L1	330,697	C	T
L2	2,621,027	A	G
L2B1	354,181	A	G
L2B2	1,408,904	C	T
L2B2sB1	1,853,849	T	C
L2B2sB1Gp1	1,163,826	T	C
L2B2sB1Gp2	559,637	G	A
L2B2sB2	707,292	T	C
L3	2,557,840	T	T
L3B1	309,945	T	C
L3B2 ***	1,767,871	A	A
L3B3	135,971	T	C
L3B3sB1	155,657	T	C
L3B3sB2	1,560,255	A	G
L3B3sB3	922,706	T	C

* Genomic position according to the reference genome of *B. mallei* ATCC 23344. ** Base that should be present in a particular genomic region for an isolate to be defined as the corresponding lineage. *** *B. mallei* BAC 86/19 is identified as lineage 3, sublineage 2.

## Data Availability

The genome assembly is available at https://www.ncbi.nlm.nih.gov/data-hub/genome/GCA_024748885.1/ (accessed on 5 October 2023). Reads are available at https://www.ncbi.nlm.nih.gov/sra/SRX15672395 (accessed on 5 October 2023).

## Supplementary Materials

The following supporting information can be downloaded at: https://www.mdpi.com/article/10.3390/pathogens12101250/s1, Table S1: Classification of *Burkholderia mallei* BAC 86/19 strain by MALDI-TOF MS.
