# Supplementary material for: Glanders Diagnosis in an Asymptomatic Mare from Brazil: Insights from Serology, Microbiological Culture, Mass Spectrometry, and Genome Sequencing
Source: Pathogens. 2023 Oct 17;12(10):1250. doi: 10.3390/pathogens12101250 (PMC10609850; doi:10.3390/pathogens12101250)
Supplement: Supplementary file 1 [file pathogens-12-01250-s001.zip › pathogens-2617187-supplementary.pdf]

Table S1. Classification of *Burkholderia mallei* BAC 86/19 strain by MALDI-TOF MS.

Analyte Name	Organism (best match)	Score Value	Organism (second best match)	Score Value
07/07	*B. mallei* BAC 86/19	2.501	*B. pseudomallei* 2356	2.039
07/07	*B. mallei* BAC 86/19	2.527	*B. mallei* 010	2.101
P2BM	*B. mallei* BAC 86/19	2.502	*B. pseudomallei* 2356	2.062
P2BM	*B. mallei* BAC 86/19	2.395	*B. pseudomallei* 2356	2.058
P2BM	*B. mallei* BAC 86/19	2.327	*B. pseudomallei* 2356	2.001
AS	*B. mallei* BAC 86/19	2.602	*B. pseudomallei* RKI	2.083
AS	*B. mallei* BAC 86/19	2.524	*B. pseudomallei* RKI	2.143
AS	*B. mallei* BAC 86/19	2.502	*B. pseudomallei* RKI	2.105
B2F	*B. mallei* BAC 86/19	2.492	*B. pseudomallei* 2356	2.069
B2F	*B. mallei* BAC 86/19	2.568	*B. mallei* Ore	2.067
B2F	*B. mallei* BAC 86/19	2.566	*B. mallei* Ore	2.114
B2M	*B. mallei* BAC 86/19	2.342	*B. mallei* Ore	2.051
B4F	*B. mallei* BAC 86/19	2.565	*B. pseudomallei* RKI	2.246
B4F	*B. mallei* BAC 86/19	2.614	*B. pseudomallei* RKI	2.245
B4F	*B. mallei* BAC 86/19	2.536	*B. mallei* Flash	2.108
B4F	*B. mallei* BAC 86/19	2.558	*B. pseudomallei* RKI	2.252
B4F	*B. mallei* BAC 86/19	2.581	*B. pseudomallei* RKI	2.337
B4M	*B. mallei* BAC 86/19	2.577	*B. mallei* Ore	2.089

B4M	*B. mallei* BAC 86/19	2.619	*B. mallei* Ore	2.197
M15	*B. mallei* BAC 86/19	2.514	*B. pseudomallei* RKI	2.229
M15	*B. mallei* BAC 86/19	2.508	*B. pseudomallei* RKI	2.113
M15	*B. mallei* BAC 86/19	2.584	*B. pseudomallei* RKI	2.246
M16	*B. mallei* BAC 86/19	2.338	*B. mallei* 176	2.056
M2-11	*B. mallei* BAC 86/19	2.518	*B. pseudomallei* RKI	2.273
M2-11	*B. mallei* BAC 86/19	2.56	*B. mallei* Ore	2.101
M2-11	*B. mallei* BAC 86/19	2.55	*B. pseudomallei* RKI	2.163
M4-14	*B. mallei* BAC 86/19	2.343	*B. mallei* ATCC 23344 RKI	2.066
M7-18	*B. mallei* BAC 86/19	2.547	*B. mallei* Flash	2.18
M7-18	*B. mallei* BAC 86/19	2.588	*B. mallei* Ore	2.216
M8-19	*B. mallei* BAC 86/19	2.509	*B. mallei* Flash	2.26

B. mallei 86/19 - MSP generated in this work, as described in Material and Methods section;
B. mallei ATCC 23344 RKI and *B. pseudomallei* RKI - MSPs generated as described (Verbisck et al., 2020) using open access mass spectra from RKI - Robert Koch Institute, Berlin, Germany (Lasch et al., 2016);
B. mallei 010, 176, Ore and Flash and *B. pseudomallei* 2356 - mass spectra and MSPs generated as described (Verbisck et al., 2020), from clinical strains isolated at LFDA-MG - Federal Agricultural Defense Laboratory of Minas Gerais, Brazil.
